# Books and Magazines

**Published:** 1911-02

**Authors:** 


### Books and Magazines.

Vecki's Prevention of Sexual Diseases. Critic & Guide, Publishers, New York. Price, $1.50.

This book is written by an earnest student of the sexual and venereal problem,—in a deep scientific spirit—and while it is on a "delicate" subject—owing to our shamefaced false modesty, there is nothing in it to shock the most prudish. The discussion of this subject is now very general, and the only hope of reform lies in public enlightenment. To this end, Dr. Vecki's little book will contribute. Get it. Lend it to your clients.

Diagnosis and Treatment of Diseases of Women. By Harry Sturgeon Crossen, M. D., Professor of Clinical Gynecology, Washington University; Gynecologist to many of the St. Louis hospitals, etc. Second edition, revised and enlarged, with seven hundred and forty-four engravings. St. Louis: C. V. Mosby Company. 1910. Cloth, $6.00.

This is a standard text-book. Whatever is new in obstetrics and gynecology—since last edition—has been incorporated. It is like Gray's Anatomy,—it is standard. The general principles are unchanged and there is nothing new in diagnosis, and one may say the last word has been said on the subject of diagnosis and treatment. The publishers have done themselves credit in the mechanical get-up of this excellent work.

---

The Physician's Visiting List (Lindsay & Blakiston's) for 1911. *Sixtieth year* of its publication. With special memoranda, 25 patients a week. Interleaved. Price, $1.25. P. Blakiston's Son & Co., Philadelphia.

This old favorite is too well known to warrant any words of commendation here and now. Every doctor knows its value and their grand-daddies knew it before them. It is standard as the

U. S. P. Nice soft morocco and fine paper, clear type and gilt-edge. I can't imagine a more appropriate New Year's gift for a friend. Send for a copy.

---

PRINCIPLES OF PUBLIC HEALTH. By Thomas D. Tuttle, B. S., M. D., State Board of Health, Montana. Published by the World Book Company—is along the same lines. The title expresses its object; for use in schools.

---

PRIMER OF SANITATION.—Primer of Hygiene, New World Series, the Only School Book of its Kind,—a Brief. By Ritchie & Caldwell. World Book Company, Yonkers, New York. The Brief is a lot of testimonials from teachers,—a pamphlet. The Primer of Hygiene is a new edition of the work which obtained such an extensive sale last year, and which was reviewed in the Red Back. Its purpose is sufficiently set forth in the title. Every pupil in all the schools should be taught the principles of hygiene, and we hope to see this excellent primer used everywhere—if we would grow a race of healthy people. $1.00

